# Supplementary material for: Rational strain design with minimal phenotype perturbation
Source: Nat Commun. 2024 Jan 24;15:723. doi: 10.1038/s41467-024-44831-0 (PMC10808392; doi:10.1038/s41467-024-44831-0)
Supplement: Supplementary file 1 — Supplementary Information [file 41467_2024_44831_MOESM1_ESM.pdf]

Rational strain design with minimal phenotype perturbation

Narayanan *et al.*

Supplementary Note 1. Phenotype perturbation sensitivity analysis

In the main text, we qualitatively demonstrated the importance of constraining the deviation of the engineered strains from the reference phenotype (Figure 3, main text) using the kinetic models K_trpD9923. We accomplished this by varying the allowable fold changes in concentrations while fixing the number of perturbed enzymes (3 enzymes) and the maximum permissible fold changes in enzyme activities (5-fold).

We extend this result by conducting a quantitative sensitivity analysis across 48 different combinations of allowable fold changes in enzyme activity (2, 3, 5, and 10-fold) and allowable fold changes in concentrations (2, 3, 4, 5, 6, 7, 8, 9, 10, 12, 15, and 20-fold). For all the combinations, we permitted a maximum of 3 enzymatic interventions.

For a given combination of maximal enzymatic fold change and permissible deviation from reference phenotype (concentration fold change), we generated the top design for each of the 10 kinetic models in K_trpD9923 using NRA. Since the log-linear approximation is accurate for smaller changes in enzyme activities and may be inaccurate for larger changes in enzyme activity, we also conducted the nonlinear simulations in the batch reactor setup as the relationship between enzyme activity and phenotype becomes more complex and nonlinear. Considering that in industrial fermentation settings, next to the titer, the time taken to reach the final product titer is also essential, we utilized the production rate to compare different combinations. We define the production rate as the final anthranilate titer divided by the total production time, where the total production time is measured at the time point when the final titer is achieved.

We applied each top design to the corresponding model, simulated its behavior in a batch fermentation setting, and stored the production rate. We then recorded (i) the mean NRA predicted increase in anthranilate yield and (ii) the mean simulated production rate across the 10 designs for all 48 combinations of allowable enzymatic and concentration fold changes.

As expected, the mean NRA predicted increase in anthranilate production across the 10 models increases as we permit a more significant deviation from the reference phenotype and a greater fold change in enzyme activity for each of the 3 target enzymes (Supplementary Figure 1a). This

predicted increase is based on a *log-linear approximation* of the system's nonlinear behavior around the reference steady state.

Our results from the nonlinear simulations show the need to balance flexibility and robustness (Supplementary Figure 1b). We observe that across all allowable enzyme activity levels, there is a decline in the mean production rate across the 10 models as we move further than 10-fold permissible concentration change away from the reference phenotype. Moreover, for 2, 3, and 5-fold allowable changes in enzyme activity, the best-performing designs are obtained when the engineered strains are constrained to be close to the reference phenotype (less than 4-fold permissible changes in concentrations).

Moreover, the extent to which we can deviate from the reference phenotype depends on the strength of the enzymatic manipulations. Under more substantial interventions (5-fold and 10-fold changes), mean productivity drops more significantly (18% and 52%) between the engineered strains that are closest and furthest to the reference phenotype. On the other hand, under slight enzymatic modifications (2-fold and 3-fold enzyme activities), there is a smaller difference between the designs generated with the most and the least stringent constraints on proximity to the reference strain (10% and 7%). Our hypothesis for this is that the designs with smaller fold changes in enzyme activity alter the phenotype of the reference strain to a lesser extent, which is supported by the plateau in production rates observed at higher allowable deviations (>10-fold changes in concentrations). Taken together, these findings suggest that when aiming to develop reliable designs with significant enzymatic interventions, it is strongly advised to be cautious and limit the extent to which the engineered strains deviate from the reference phenotype.

Supplementary Note 2. MCA design

In the main text, we demonstrated the drawbacks of conducting strain design using the top yield control coefficients (CCs) for anthranilate with respect to glucose uptake without imposing any constraints on the phenotype perturbation. In practice, an engineer would likely use their expert knowledge to judiciously choose those control coefficients that do not have an adverse impact on

the network. To understand the implications of such an approach, we chose the top 3 yield CCs for each kinetic model in K_trpD9923 that did not reduce the growth rate. We did this by screening for enzymes with the same qualitative control (positive or negative) over both anthranilate and biomass yield. We then selected the top 3 enzymes among the filtered enzymes based on their anthranilate yield CC and applied them using a 5-fold perturbation. We observed that even with this choice of targets, both growth and anthranilate production were severely hindered (Supplementary Figure 2). Indeed, it is only when we limit the allowable fold change in perturbations of enzyme activity to less than 1.5 fold (Supplementary Figure 4) that the designs start yielding superior anthranilate titers. This further supports NRA as a holistic, systematic approach to applying design constraints.

Supplementary Note 3. Alternative routes for producing anthranilate in K_trpD9923

We conducted a clustering analysis of the 41 unique designs (Figure 4, main text) generated using K_trpD9923 to detect common patterns and routes toward producing anthranilate across the designs. This revealed the presence of five clusters of alternative enzymatic interventions satisfying the imposed design specifications. All the designs redirect carbon to the shikimate pathway by increasing the activity of DDPA, which serves as the entry point to the pathway. The clusters differed by the choice of the other two target enzymes. Cluster I consists of two designs, one of which concentrates the flow of carbon through the shikimate pathway (CHORS and DHQS), while the other increases the activity of anthranilate synthase (ANS) and the activity of NADH5 in the electron transport chain (ETC). Cluster II has three designs, all of which reduce the activity in the Krebs cycle (FUM). Two of the designs also increase the availability of glutamine, which is a substrate for ANS, either by increasing its synthesis (GLNS) or decreasing the conversion of glutamate to α-ketoglutarate (GLUDy) so that it is available for glutamine synthesis. The third design increases the activity of SHKK in the shikimate pathway.

Cluster III contains designs that all target the availability of glutamate for glutamine synthesis by reducing its degradation (GLUDy). Some of the designs also balance the availability of the precursor metabolites, e4p and pep, by targeting enzymes in the PPP or glycolysis. The others

target either the activity in the shikimate pathway (SHKK, ANS) or the availability of glutamine (GLNS).

The largest cluster, cluster IV, has designs that focus on the shikimate pathway by increasing the activity of SHKK. Additionally, in a manner similar to cluster III, some designs in this group ensure the balance between the two shikimate pathway precursors by targeting glycolysis (ENO, FBA, etc.) or PPP (TALA, RPI). The remaining designs target either growth, through the ETC (SUCDi), the Krebs cycle (ICDHyr, AKGDH), or anaplerotic reactions (ME2, ICL), or the production of anthranilate through the shikimate pathway (DHQS) (Figure 4, main text).

Finally, cluster V is an agglomeration of designs that focus on glutamine synthesis with all but one of the designs targeting GLNS. Additionally, the designs target enzymes in the shikimate pathway (SHKK, SHK3Dr), the ETC chain (SUCDi, ADK1), the Krebs cycle (ACONTa) and glycolysis (FBA).

Supplementary Note 4. Addendums to Figure 5

The growth and glucose curves for the simulated experimental designs and the NOMAD proposed designs are shown in Supplementary Figure 5. As expected, the growth dynamics is significantly closer to the reference strain than what was observed experimentally (Figure 3 and Table 1 of the experimental work [1]).

Supplementary Note 5. Investigating inferior titers of K_trpD9923_d2

As part of the validation for our models, we implemented two previously reported experimental designs in each of the 10 kinetic models, K_trpD9923 (Figure 5, main text). While our in-silico strains, K_trpD9923_d1 and K_trpD9923_d2, captured the experimentally observed trends, they were unable to reproduce the exact titers that were obtained. In particular, K_trpD9923_d2 produced titers of around 0.4 g/L while the experimental equivalent, W3110 trpD9923/pJLaroG^{fbr}tktA, attained titers of 0.75$\pm$0.04 g/L in actual experiments.

We used NRA to identify what additional enzymatic changes could enable us to achieve the experimentally observed titers. To do this, we allowed up to 7 enzymatic targets, of which DDPA,

TKT1, and TKT2 were mandatory choices. We also permitted a maximum of 5-fold change in concentrations and enzyme activity levels. With these constraints, we generated the top designs for each of the 10 kinetic models and collated them. For each model, the stopping criterion for design generation was when the predicted increase in anthranilate was less than 99% of the maximal value.

The 26 generated designs encompassed 17 different enzymes in addition to DDPA, TKT1 and TKT2, of which 5 were in the Shikimate pathway. The most frequently appearing design (6/10 models) was the one that targeted GLUDy, SHKK, DHQS, and ANS in addition to DDPA, TKT1 and TKT2. We tested this design in bioreactor simulations using the mean NRA predicted fold changes for each enzyme, in addition to deregulating DDPA. Remarkably, we could recover median anthranilate titers of around 0.7 g/L (Supplementary Figure 6a) which is in line with the experimental observations. We also observed a drop in growth in our in-silico strains, from 1.2g/L (Supplementary Figure 5a) to 1.05 g/L (Supplementary Figure 6b) which is more comparable to the experimentally observed growth of 0.93$\pm$0.04 g/L.

This experiment highlights the utility of NOMAD in kinetic modeling and strain design endeavors. By using information inherent to kinetic models, it can identify the sources of discrepancies between simulations and new data coming from additional experiments, thereby lending itself naturally to DBTL cycles.

Supplementary Note 6. Prioritizing designs for experimental implementation

We used a two-stage process involving log-linear approximations and nonlinear bioreactor simulations to prioritize the designs generated using K_trpD9923 for experimental implementation. In the first stage, we analyzed the robustness of the 41 unique designs generated for K_trpD9923 by applying them to all the models in an NRA setting and calculating the maximum predicted anthranilate yield under the usual constraints - maximum 5-fold upregulation, unlimited downregulation, maximum 3-fold concentration change, and maximum 3-enzyme modifications (Supplementary Figure 7a). We then ranked the designs based on their average predicted increase in anthranilate yield across the 10 models. The top 5 designs all targeted DDPA and GLUDy, varying

only by the third target enzyme (Supplementary Figure 7b). The mean NRA proposed fold changes in enzyme activity for the top 5 designs are given in Supplementary Table 1.

The second stage of the ranking process consists of testing designs using simulated batch fermentations that closely replicate real-world conditions (Methods). We evaluated the 5 designs that had the highest mean NRA predicted increase in anthranilate yield across the K_trpD9923 models. The nonlinear simulations incorporated the enzyme activity fold changes recommended by NRA for each model. The top four designs (d-1 – d-4) were tied in ranking and performed well across the phenotypic uncertainty covered by the 10 models. They remained close to the phenotype of the reference strain while providing >25% increase in anthranilate titers, as shown by the mean of their responses across the 10 models (Supplementary Figure 8a). Design d-5 (DDPA, GLUDy, ANS) was discarded due to its poor performance across the models - it displayed significantly slower dynamics and only reached the anthranilate titers of the reference strain after 40 hours.

Given that DDPA and GLUDy appeared in all the five designs with the highest mean NRA predicted increase in anthranilate, we also studied the response of the models when we only applied the double mutant without the third enzyme. The results of this study are presented in Supplementary Note 7.

The experimental implementation of the top four designs could deviate from the NRA-suggested fold changes in enzyme activities. We evaluated the margin for error afforded by the four designs (d-1 – d-4) by studying their sensitivity to perturbations in the mean NRA-proposed fold changes in enzyme activity. We found that all four designs could withstand errors in experimental implementation, retaining their performance when subjected to a $\pm 50\%$ perturbation to all three enzymes together (Supplementary Figure 8c) or to each enzyme individually (Figures S.8d-f). By retaining their performance across a range of models, and a spread of enzyme expression levels, the four screened designs, DDPA + GLUDy + PGI/GND/HEX1/PYK, proved to be robust to physiological and expression level uncertainties and can thus be confidently passed on for experimental validation.

Supplementary Note 7. Double Mutant (DDPA + GLUDy) in K_trpD9923

The top 5 designs for improving anthranilate in K_trpD9923 all recommend the upregulation of DDPA and the downregulation of GLUDy, while varying by the third target enzyme. Given that DDPA and GLUDy are regularly present, our objective was to investigate the consequences of solely manipulating the activity of these two enzymes. To do this, we applied a 4-fold upregulation of DDPA and a 1.2-fold downregulation of GLUDy to all the 10 kinetic models, in line with the mean NRA suggested fold changes for the two enzymes. The engineered strains reach a median titer of 0.41 g/L (Supplementary Figure 9), which is in line with the designs that target three enzymes simultaneously (Supplementary Figure 8b). In terms of growth, the double mutant produced a median biomass titer of 1.278 g/L while the top 4 triple mutants had mean biomass titers of 1.26 – 1.29g/L. From the relatively similar performances of the double and triple mutants, it appears that DDPA and GLUDy carry the bulk of the work in terms of redirecting resources toward anthranilate production. This titer comes at a negligible cost to growth.

Supplementary Note 8. Alternate routes for overproducing anthranilate in eK_trpD9923 and eK_trpD9923_d2

We analyzed the designs generated using the enhanced models to identify trends and clusters in them. There were 34 unique designs generated for eK_trpD9923, encompassing 34 different enzymes. These designs were broadly grouped into two clusters (Supplementary Figure 10). Cluster I had four designs that all upregulated DDPA in the Shikimate pathway while downregulating at least one enzyme in glycolysis. The third enzyme was used to further downregulate activity in glycolysis, or to target the anapleurotic reactions (PPCK: phosphoenolpyruvate carboxykinase) or the electron transport chain (SUCDi: Succinate dehydrogenase). Cluster II contained 30 designs, all of which targeted ANS and DDPA simultaneously. The third enzyme suggested balancing the availability of the precursors pep, and e4p by targeting the other central carbon pathways.

We similarly identified 13 unique designs for improving the anthranilate yield in eK_trpD9923_d2. These designs were clustered into three groups of similar designs (Supplementary Figure 11). All

groups suggest upregulating activity in the Shikimate pathway by targeting at least two enzymes in the pathway. The groups varied by the choice of the two enzymes in the Shikimate pathway, and the additional pathway that is targeted by the designs. The five designs in group I all target anthranilate synthase (ANS) and 3-dehydroquinate synthase (DHQS). They all vary by the third enzyme which either reduces activity through glycolysis or the TCA cycle (PGK: phosphoglycerate kinase / AKGDH: alpha-keto glutarate dehydrogenase), increases the availability of glutamate for glutamine synthesis by downregulating glutamate dehydrogenase (GLUDy), or targets the anapleurotic reactions (PPC: phosphoenolpyruvate carboxylase / ICL: isocitrate lyase).

Cluster II contains three designs that all upregulate ANS activity and downregulate PPC. The third enzyme either downregulates glycolysis (PGI: glucose-6-phosphate isomerase), increases the availability of glutamine (GLUDy) or targets the shikimate pathway further through the upregulation of shikimate kinase (SHKK).

Cluster III contains five designs out of which four suggest downregulating activity through glycolysis (PGi, FBA or GAPD: Glyceraldehyde-3-phosphate dehydrogenase) and increasing activity through the shikimate pathway (DDPA, CHORS: chorismite synthase, ANS). The other design enhanced activity through the pentose-phosphate-pathway (PPP) through the upregulation of 6-phosphogluconolactonase (PGL).

It is noteworthy that there are only 13 designs that are predicted to increase the anthranilate yield in the double mutant, as opposed to 34 designs for eK_trpD9923. This suggests that as we optimize the strain, we will have fewer ways to enhance its performance.

Supplementary Note 9. Prioritizing designs from enhanced kinetic models

Designs for improving eK_trpD9923

There were 123 designs, encompassing 34 enzyme targets, that produced at least 95% of the maximal anthranilate yield for eK_trpD9923. Out of these designs, 34 were unique by membership. We analyzed the robustness of these unique designs based on their predicted log-linear performance across all the models, and chose the five designs (Supplementary Table 2) with

the highest mean predicted increase in anthranilate yield for further inspection in a bioreactor setting. These designs all suggested upregulating DDPA and ANS. They varied based on the third target enzyme - FBA (d-1) / GAPD (d-2) / PGK (d-3) / G6PDH2r (d-4) / PGL (d-5).

All five designs demonstrated their robustness to the choice of kinetic model by producing higher mean titers (0.76 – 0.81 g/L) than the best performing experimental strain, W3110 trpD9923/pJLaroG^{fbr}tktA which produced 0.75 g/L (Supplementary Figure 12b).

Next, we evaluated the sensitivity of these designs to 50% perturbations in the maximal velocities of the involved enzymes (see Methods). The performance of the designs was compromised when all three enzymes were perturbed simultaneously (Supplementary Figure 12c); they displayed slower dynamics of anthranilate production after 20 hours. The perturbation analysis of the individual enzymes revealed that this reduced performance was caused by the sensitivity of the designs to incorrect implementations of ANS (Supplementary Figure 12d). Interestingly, this observation corroborated the evidence from the first study where the design that involved ANS (DDPA, GLUDy, and ANS) performed poorly in the nonlinear verification study (Supplementary Note 6). The designs proved more robust to perturbations applied to the suggested enzyme activities for DDPA and the third enzyme (Supplementary Figure 12e and f). The results of these tests suggests that special care needs to be taken for ANS during experimental implementation.

Designs for improving eK_trpD9923_d2

There were 13 unique designs that were proposed by NRA for improving the anthranilate yield in eK_trpD9923_d2. We enforced these designs in eK_trpD9923_d2 using NRA and identified the 5 designs with the highest mean predicted yield in anthranilate across the models (Supplementary Table 2). These designs all proposed upregulating ANS, and either DDPA (d-1, d-2) or DHQS (d-3, d-4, and d-5). The third enzyme in each design either downregulated one of GLUDy (d-1) / GAPD (d-3) / FBA (d-4) or upregulated AKGDH (d-2) / PGL (d-5). All five designs proved robust to the choice of kinetic model, providing titers that were superior to those of the two engineering strains (Supplementary Figure 13a). Four out of the five designs maintained their final anthranilate titers even under a 50% perturbation of the enzymatic fold changes of all 3 enzymes simultaneously (Supplementary Figure 13b). Although the titers obtained under perturbation for design d-1 (ANS,

DHQS, GLUDy) was lower than the value of its unperturbed counterpart, it was still superior to the titers of the experimental strains. This suggests that all five designs are ready to be tested experimentally.

Supplementary Note 10. Top 4 designs from K_trpD9923 perform well in eK_trpD9923

The superior performance of the designs generated using eK_trpD9923 prompted us to verify the performance of the top 4 designs from the first study (DDPA, GLUDy, and PGI/GND/HEX1/PYK) in these enhanced models. We ensured a fair evaluation of the four designs by enforcing their enzyme membership in each of the 13 models in eK_trpD9923 under the same constraints that were used to generate the new NRA designs – 10-fold and 2.5-fold permissible changes in enzyme activities and concentrations. We then took the mean of the suggested fold changes in enzyme activity for each design and applied them to eK_trpD9923 in bioreactor simulations. The median anthranilate titers of the 4 designs from K_trpD9923 (0.68 g/L) were lower than those of the top 5 designs from eK_trpD9923 (0.78 g/L) across the 13 models (Supplementary Figure 14a). However, they exhibited a closer adherence to the dynamics of the reference strain, reaching their maximum titers in 18 hours as opposed to 22 hours for the top 5 designs generated using eK_trpD9923. This result indicates that the 4 designs produced with K_trpD9923 remain suitable for experimental validation.

Supplementary Note 11. Reaction Information

Information about the rate laws and regulatory information used for each reaction are provided in the Supplementary Data 1 and 2. The impact of inhibitors (I) and activators (A) were modeled as shown below for a sample Michaelis-Menten equation with one substrate (S) and one product (P). Further information on the way reaction mechanisms are modeled can be found in the supplementary information section of SKiMPy [2]. Regulatory information for all reactions except for two, ANS [3] and DDPA[4], were obtained from an earlier kinetic modeling study[5].

a) Simple inhibition:

$$v = V_{\max} \cdot \left(1 - \frac{\Gamma}{K_{\mathrm{eq}}}\right) \cdot \left(\frac{\frac{S}{K_S^M}}{\frac{S}{K_S^M} + \frac{P}{K_P^M} + 1}\right) \cdot \left(\frac{\mathbf{1}}{\mathbf{1} + \frac{\boldsymbol{I}}{\boldsymbol{K_I}}}\right) \quad (\mathbf{1})$$

b) Competitive inhibition:

$$v = V_{\max} \cdot \left(1 - \frac{\Gamma}{K_{\mathrm{eq}}}\right) \cdot \left(\frac{\frac{S}{K_S^M}}{\frac{S}{K_S^M} + \frac{P}{K_P^M} + 1 + \frac{\boldsymbol{I}}{\boldsymbol{K_I}}}\right) \quad (\mathbf{2})$$

c) Mixed inhibition:

$$v = V_{\max} \cdot \left(1 - \frac{\Gamma}{K_{\mathrm{eq}}}\right) \cdot \left(\frac{\frac{S}{K_S^M}}{\frac{S}{K_S^M} + \frac{P}{K_P^M} + 1 + \frac{\boldsymbol{I}}{\boldsymbol{K_I}}}\right) \cdot \left(\frac{\mathbf{1}}{\mathbf{1} + \frac{\boldsymbol{I}}{\boldsymbol{K_I}}}\right) \quad (\mathbf{3})$$

d) Simple activation:

$$v = V_{\max} \cdot \left(1 - \frac{\Gamma}{K_{eq}}\right) \cdot \left(\frac{\frac{S}{K_S^M}}{\frac{S}{K_S^M} + \frac{P}{K_P^M} + 1}\right) \cdot \left(\mathbf{1} + \frac{\boldsymbol{A}}{\boldsymbol{K_A}}\right) \quad (\mathbf{4})$$

Supplementary Note 12. Dominant time constants

NOMAD uses the linearized time constants of the putative kinetic models to screen for models with physiologically relevant dynamics. Each kinetic model is represented by a system of ordinary differential equations (ODEs) that track the temporal evolution of different species in the metabolic network:

$$\frac{d\mathbf{x}}{dt} = f(\mathbf{x}, \mathbf{p}, t) = \mathbf{N} \cdot \mathbf{v}(\mathbf{x}, \mathbf{p}, t) \quad (\mathbf{5})$$

where $\mathbf{N}$ is the stoichiometric matrix, $\mathbf{x}$ is the vector representing the different metabolite concentrations, and $\mathbf{p}$ is the vector of kinetic parameters that characterize the system. We can linearize this system around its steady state by calculating its Jacobian, $\boldsymbol{J} = \left[\frac{\partial}{\partial x_1} f(\mathbf{x}, \mathbf{p}, t) \quad \dots \quad \frac{\partial}{\partial x_n} f(\mathbf{x}, \mathbf{p}, t)\right]$, which is the matrix of the 1st-order partial derivatives of $f(\mathbf{x}, \mathbf{p}, t)$ at the steady state $\bar{\mathbf{x}}$. Given that at the steady state we have that $\frac{d(\bar{\mathbf{x}})}{dt} = f(\bar{\mathbf{x}}, \mathbf{p}, t) = \mathbf{0}$, we obtain

$$\frac{d(\bar{\mathbf{x}} + \boldsymbol{\Delta}\mathbf{x})}{dt} \cong f(\bar{\mathbf{x}}, \mathbf{p}, t) + \mathbf{J} \cdot \boldsymbol{\Delta x}$$

$$\Rightarrow \frac{d\boldsymbol{\Delta x}}{dt} = \mathbf{J} \cdot \boldsymbol{\Delta x} \quad (\mathbf{6})$$

where $\boldsymbol{\Delta}\mathbf{x}$ represents the vector of infinitesimal perturbations to the metabolite concentrations. To analyze the stability of the linearized system (**6**) we can calculate the eigenvalues λ_i and eigenvectors $\mathbf{w_i}$ of the Jacobian that satisfy:

$$\lambda_i \mathbf{w_i} = \mathbf{J} \cdot \mathbf{w_i} \quad (\mathbf{7})$$

A system that is locally stable at a steady state will restore itself to that steady state in response to infinitesimal perturbations, and all its eigenvalues will have negative real parts. In simpler terms, when the system experiences perturbations, it will display aperiodic or oscillatory responses that decay exponentially at different timescales [6]. In the context of biologically relevant models, it is crucial for these responses to settle before cell division occurs. We set different constraints for the two studies to satisfy this requirement. We required the responses to reach 99.3% of their original steady-states within five time constants for K_trpD9923, and

95% of their steady-state values within three dominant time constants for eK_trpD9923. The calculations for K_trpD9923 are presented below.

The pools (y_i) of metabolites associated with each eigenvector $\mathbf{w_i}$ decay at a rate dictated by their corresponding eigenvalues:

$$y_i = \sum w_{ij} x_j \tag{8}$$

Eigenvalues with larger absolute values of their real part correspond to faster dynamic responses, while eigenvalues with smaller absolute values of their real part indicate slower dynamic responses. The dominant, or slowest, time constant, $\tau_d = \frac{1}{\min_i |\mathrm{Re}(\lambda_i)|}$ corresponds to the eigenvalue with the smallest absolute value of its real part.

Let's assume, for simplicity, aperiodic responses. Therefore, the eigenvalues of the system are real, and the response of the corresponding pool is described by

$$y_i(t) = y_{i,0} e^{-\frac{t}{\tau_d}} \tag{9}$$

At $t = 5\tau_d$, we obtain

$$y_i(t)|_{t=5\tau_d} = y_{i,0} e^{-5} \approx 0.007 y_{i,0} \tag{10}$$

i.e., the slowest response of the system will return to 99.3% of its steady-state within the 5 dominant time constants. Therefore, we need to ensure that the dominant time constant is at least 5 times faster than the doubling time, t_{db}.

More specifically, from (**10**) we get

$$\frac{y_i(t_{db})}{y_{i,0}} = 0.007 = e^{-\frac{t_{db}}{\tau_d}} \Rightarrow \tau_d = -\frac{t_{db}}{\ln 0.007} \Rightarrow \tau_d \approx \frac{t_{db}}{5} \tag{11}$$

For a maximum in-silico growth rate of $\mu = 0.32/hr$, obtained using pyTFA after integrating exofluxomics data, the corresponding doubling time can be calculated as:

$$t_{db} = 60 \cdot \frac{\ln 2}{\mu} = 130\ mins \tag{12}$$

In this study, we set a slightly stricter requirement that models should return to 99.5% of their steady-state within the cell's doubling time. Therefore, we calculate the required dominant time constant as $\tau_{max} = \frac{130}{5.3} = 24.5\ mins$, which we rounded to 24 minutes.

Supplementary Note 13. NRA formulation used in this work

Here we outline the objective used in NOMAD. The variables are given in Supplementary Table 3, while the constraints and indices are given in Supplementary Table 4 and Supplementary Table 5 respectively.

The objective was to maximize the *increase* in yield of anthranilate with respect to glucose uptake. We implemented this by maximizing the difference between the log flux fold change for anthranilate synthase and the log flux fold change for glucose uptake.

$$\max\ F_{\mathrm{ANS}} - F_{\mathrm{GLCtex}} \tag{13}$$

This objective is equivalent to increasing the yield of anthranilate, $\tilde{F}_{ANS} - \tilde{F}_{GLCtex}$, as

$$\begin{aligned} F_{\mathrm{ANS}} - F_{\mathrm{GLCtex}} &= \ln\frac{v_{\mathrm{ANS}}}{v_{\mathrm{ANS,ref}}} - \ln\frac{v_{\mathrm{GLCtex}}}{v_{\mathrm{GLCtex,ref}}} = \ln\frac{v_{\mathrm{ANS}}}{v_{\mathrm{GLCtex}}} - \ln\frac{v_{\mathrm{ANS,ref}}}{v_{\mathrm{GLCtex,ref}}} \\ \Rightarrow F_{\mathrm{ANS}} - F_{\mathrm{GLCtex}} &= \tilde{F}_{\mathrm{ANS}} - \tilde{F}_{\mathrm{GLCtex}} - \ln\frac{v_{\mathrm{ANS,ref}}}{v_{\mathrm{GLCtex.ref}}} \end{aligned} \tag{14}$$

where the yield of the reference strain, $\ln\frac{v_{\mathrm{ANS,ref}}}{v_{\mathrm{GLCtex,ref}}}$ is a constant in the optimization.

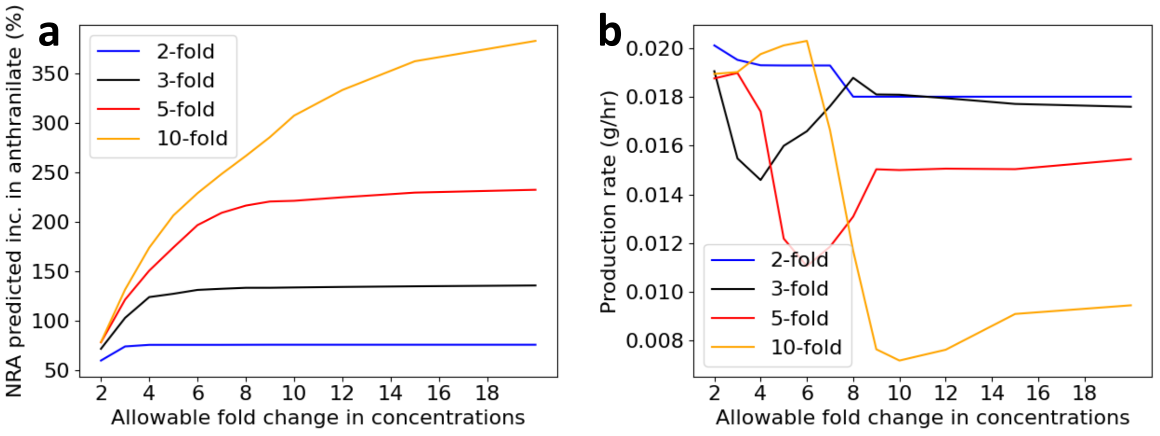

Supplementary Figure 1. Phenotype perturbation sensitivity analysis. The colored lines represent different maximal allowable fold changes in enzyme activity. For all cases, the NRA predicted more increased anthranilate production as we permit greater flexibility in the system (a). In nonlinear batch reactor simulations (b), however, how much a phenotype can be perturbed depends on the extent of the applied enzymatic interventions. The smaller the degree of intervention, the greater the leeway in constraining the deviation from the reference phenotype. Source data are provided as a Source Data file.

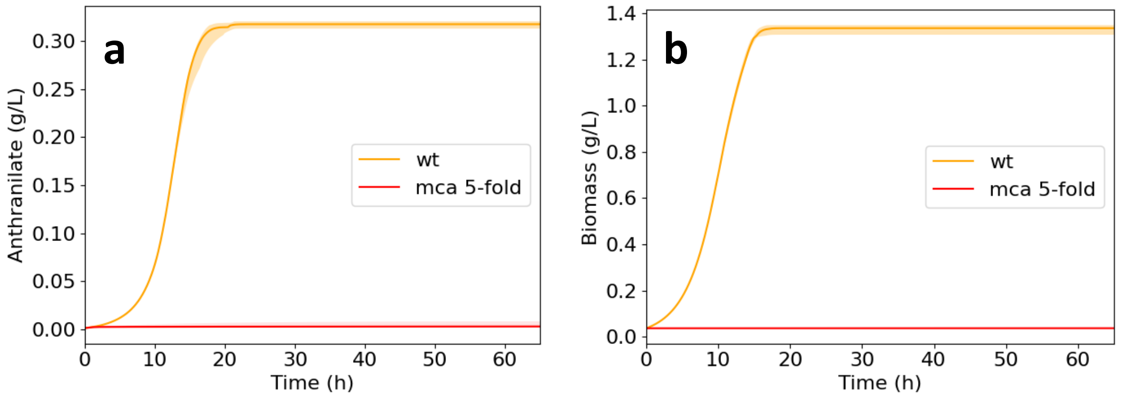

Supplementary Figure 2. Performance of MCA-based design with 5-fold changes in activity. Mean anthranilate (a) and biomass (b) curves across the 10 models for the wild type (wt) strains and MCA-designed strains when we apply a 5-fold change in enzyme activities to the top 3 anthranilate yield control coefficients that do not have an adverse effect on the growth rate. We see that both growth and anthranilate production are adversely impacted even when we account for the impact on growth rate. Source data are provided as a Source Data file.

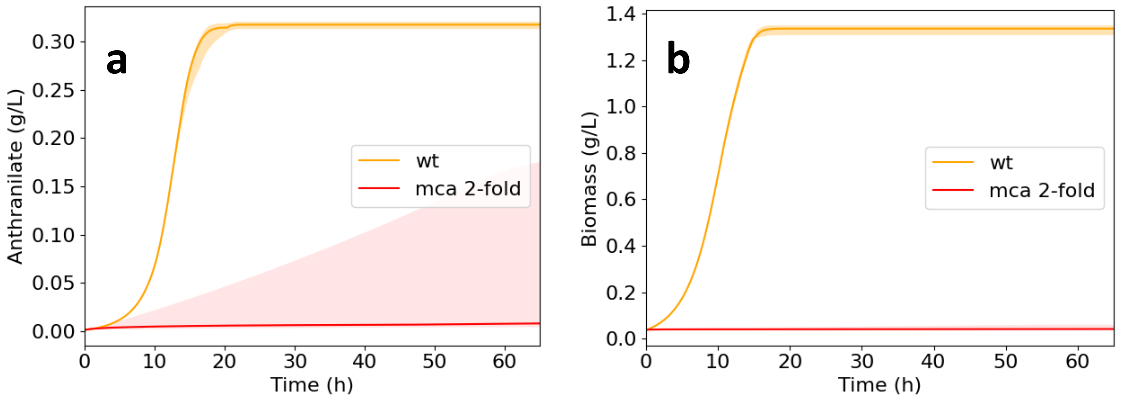

Supplementary Figure 3. Performance of MCA-based design with 2-fold changes in activity. Mean anthranilate (a) and biomass (b) curves across the 10 models for the wild type (wt) strains and MCA-designed strains when we apply a 2-fold change in enzyme activities to the top 3 anthranilate yield control coefficients that do not have an adverse effect on the growth rate. When compared with the MCA designs with 5-fold changes in enzyme activities, we see marginal improvement in anthranilate titers, although growth is still severely impacted. Source data are provided as a Source Data file.

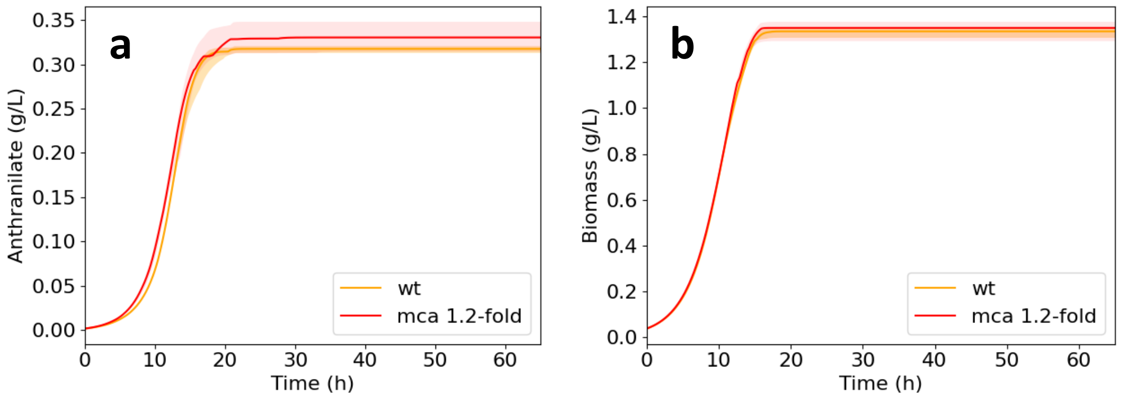

Supplementary Figure 4. Performance of MCA-based design with 1.2-fold changes in activity. Mean anthranilate (a) and biomass (b) curves across the 10 models for the wild type (wt) strains and MCA-designed strains when we apply a 1.2-fold change in enzyme activities to the top 3 anthranilate yield control coefficients that do not have an adverse effect on the growth rate. We see that as we constrain the allowable change in expression levels, a constrained MCA approach based on a judicious manual choice of control coefficients becomes more viable. Source data are provided as a Source Data file.

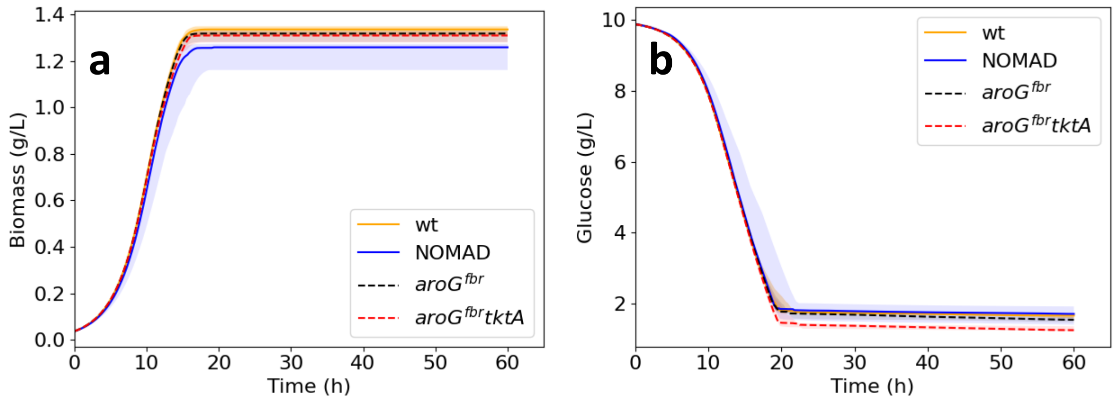

Supplementary Figure 5. Comparison of NOMAD designs vs in-silico implementations of experimental strains. Mean biomass (a) and glucose (b) curves across the 10 models when we implement the experimental strains, and when we implement the 67 NRA proposed designs in the specific models that were used to devise them. We see that the nonlinear simulations all follow the dynamics of the reference strain, with the only major change being the increase in anthranilate titers (main text). Source data are provided as a Source Data file.

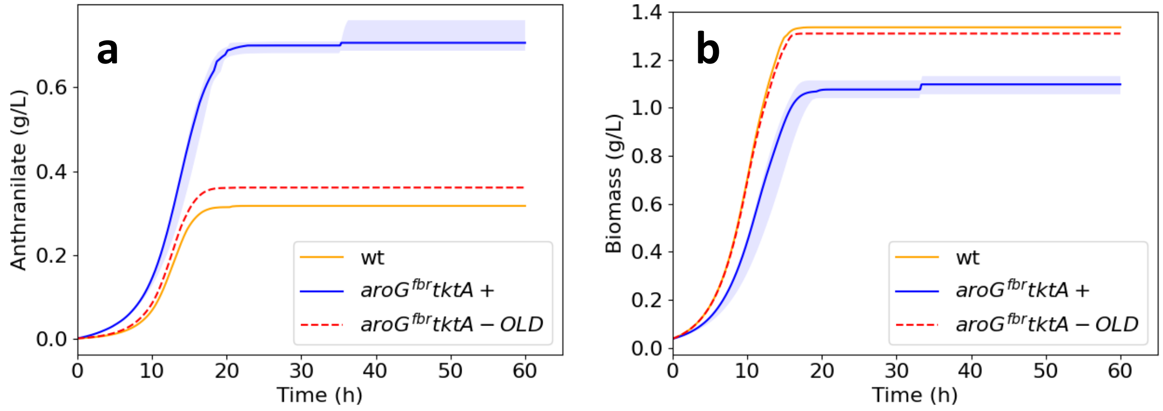

Supplementary Figure 6. Improving the accuracy of K_trpD9923_d2. Mean anthranilate (a) and biomass (b) curves across the 10 kinetic models for the reference strain (wt, solid orange), the in silico implementation of the experimental recombinant strain - trpD9923/pJLaroGfbrtktA (aroGfbrtktA-OLD, dashed red), and an augmented version of the recombinant strain (aroGfbrtktA+, solid blue) that permits 4 additional enzymes to be targeted. We see that the augmented designs produce significantly higher anthranilate titers of 0.7 g/L, at a greater cost to growth. Solid lines and shaded regions represent the mean responses and interquartile ranges across the 10 models. Source data are provided as a Source Data file.

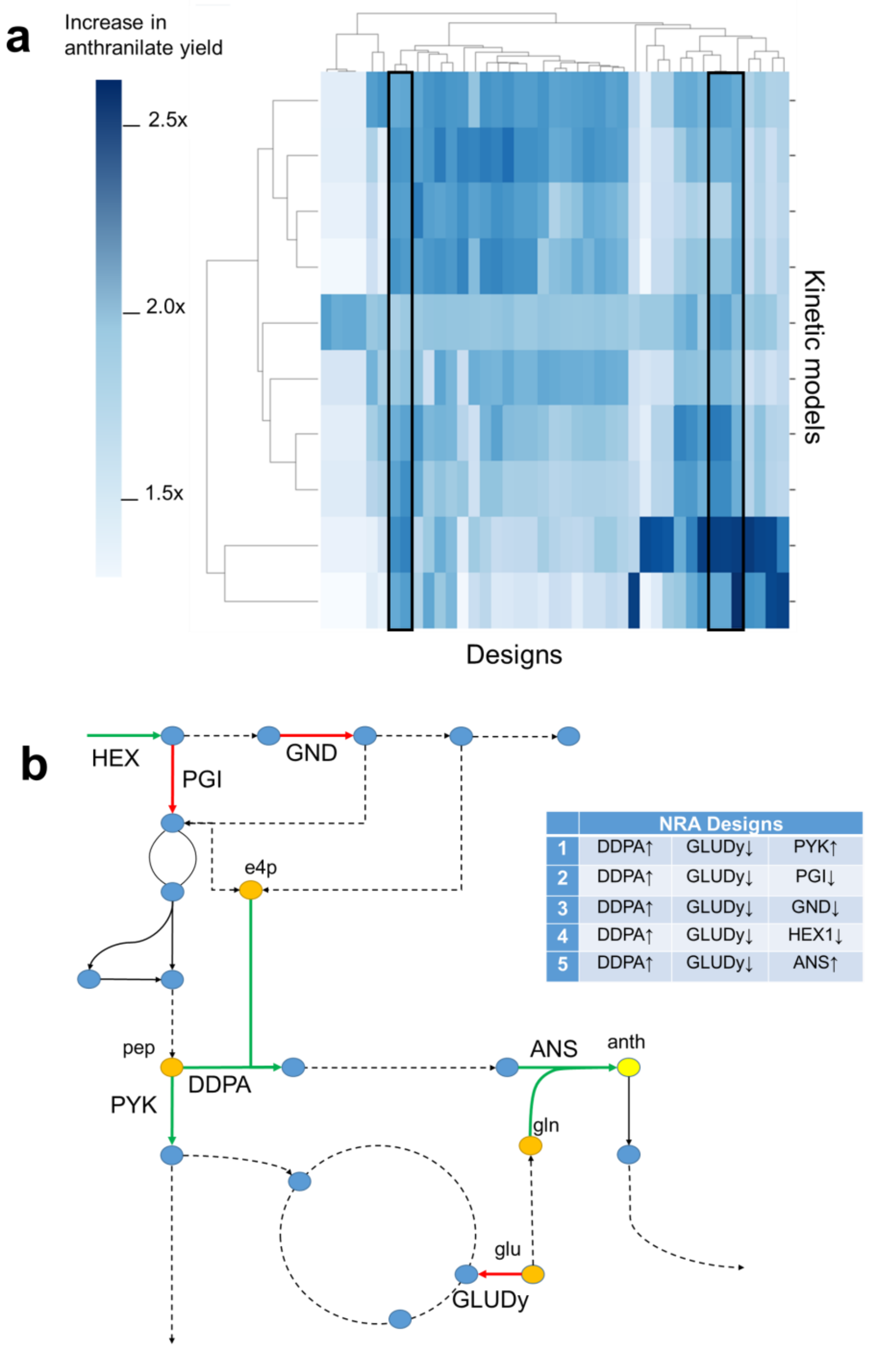

Supplementary Figure 7. Design evaluation and ranking using NRA. (a) A heat map of the predicted increase in anthranilate yield when the 41 designs (columns) are applied to the 10 kinetic models (rows), along with the five designs with the highest mean NRA solution (black rectangles). (b) A schematic of the metabolic network containing the target enzymes from these five designs. Source data are provided as a Source Data file.

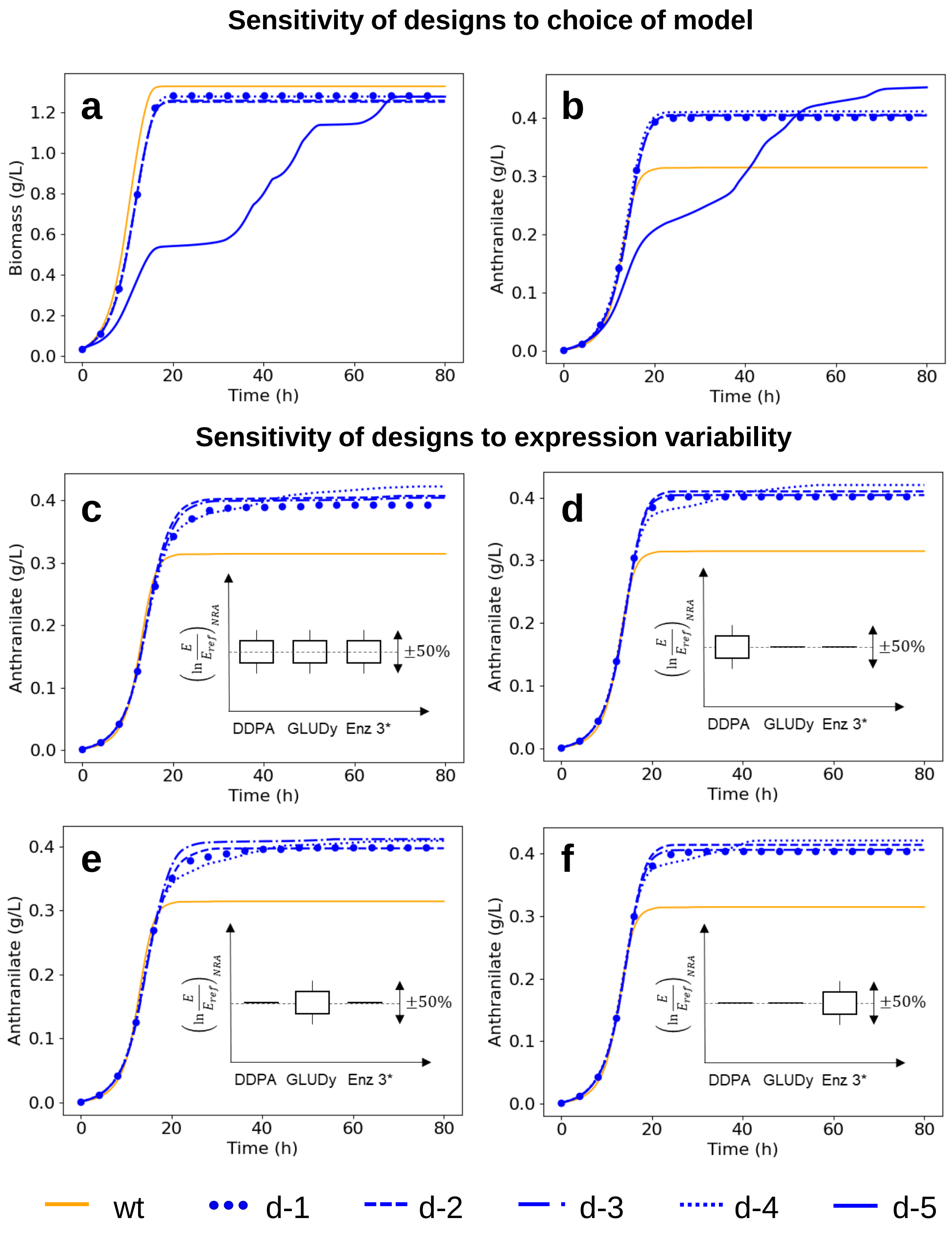

Supplementary Figure 8. Evaluation of the top 5 designs from K_trpD9923 in a fermentation setting. The mean responses across the models when the five designs are applied using the NRA suggested fold changes specific to each model (a and b). Four designs retained their performance across the models, with only d-5, targeting DDPA, GLUDy, and ANS, demonstrating altered dynamics. These four designs maintained their performance when subjected to a ±50% perturbation to their mean suggested NRA fold changes for all the enzymes (c), and for each enzyme individually (d, e, f). Source data are provided as a Source Data file.

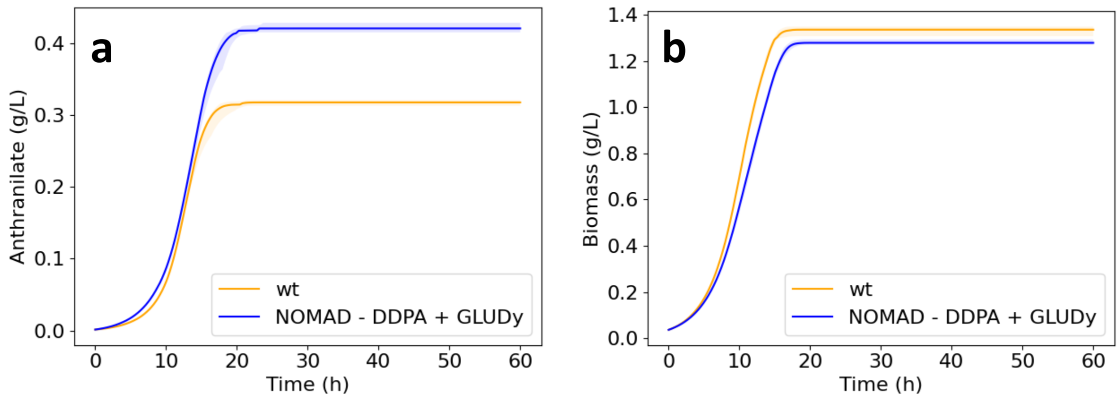

Supplementary Figure 9. Performance of the double mutant – DDPA+GLUDy. Median and interquartile ranges of anthranilate (a) and biomass (b) production when targeting DDPA and GLUDy alone. Source data are provided as a Source Data file.

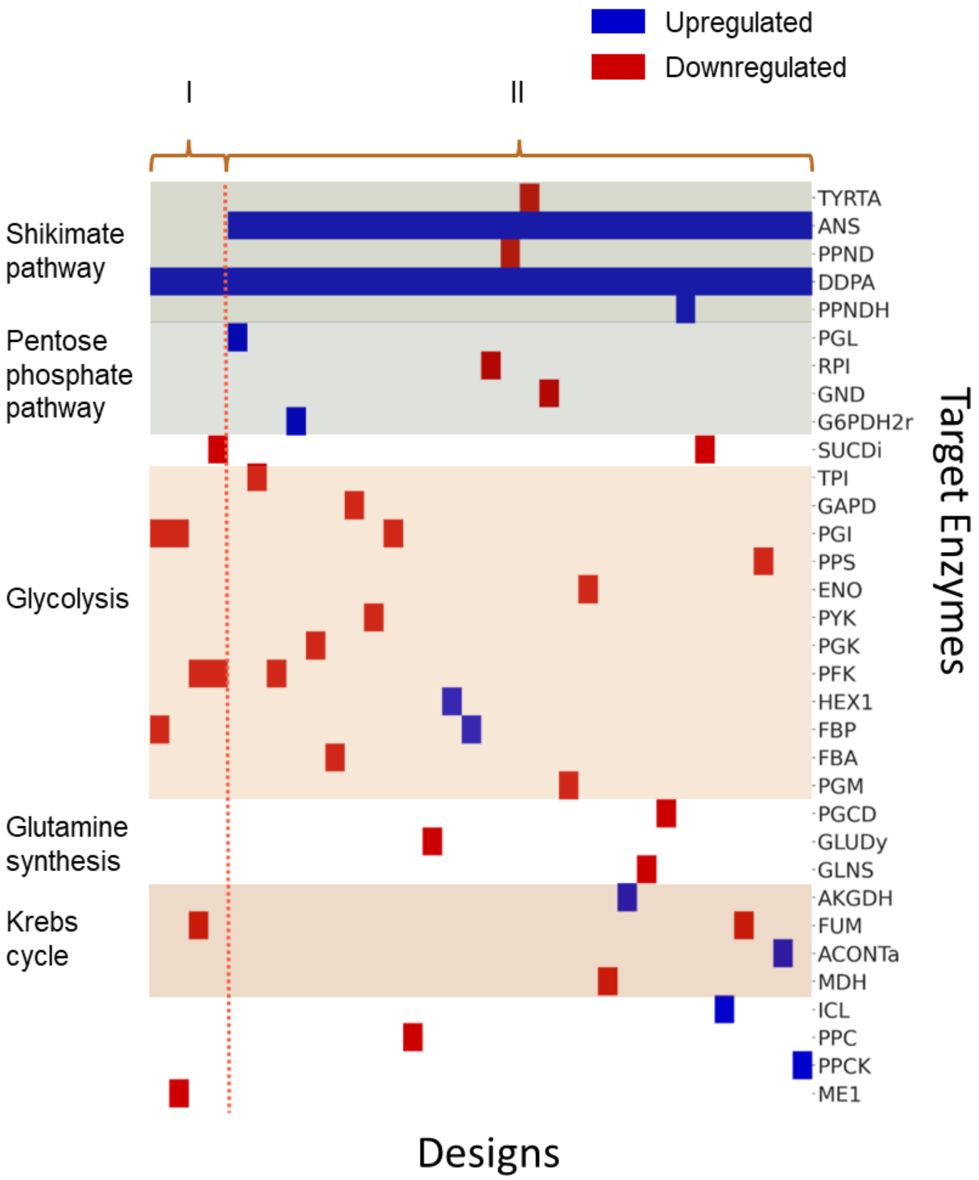

Supplementary Figure 10. Clustering analysis of NRA designs for improving anthranilate production in eK_trpD9923. The 34 unique designs encompassed 34 different enzymes and were divided into two broad groups. Source data are provided as a Source Data file.

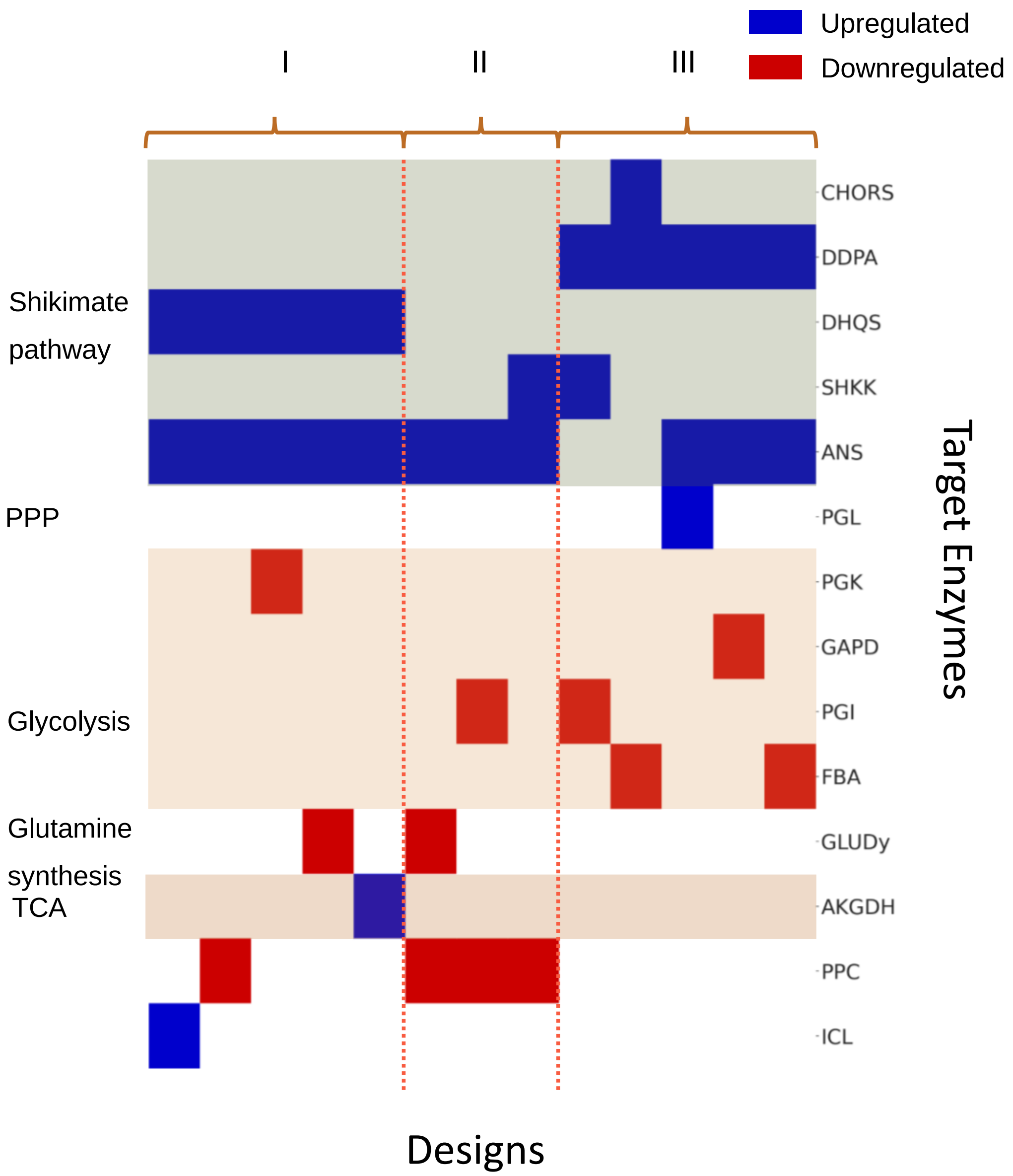

Supplementary Figure 11. Clustering analysis of the NRA designs in eK_trpD9923_d2. The 13 unique designs encompassed 14 different enzyme regulations and were divided into 3 distinct groups based on the enzymes they targeted. Source data are provided as a Source Data file.

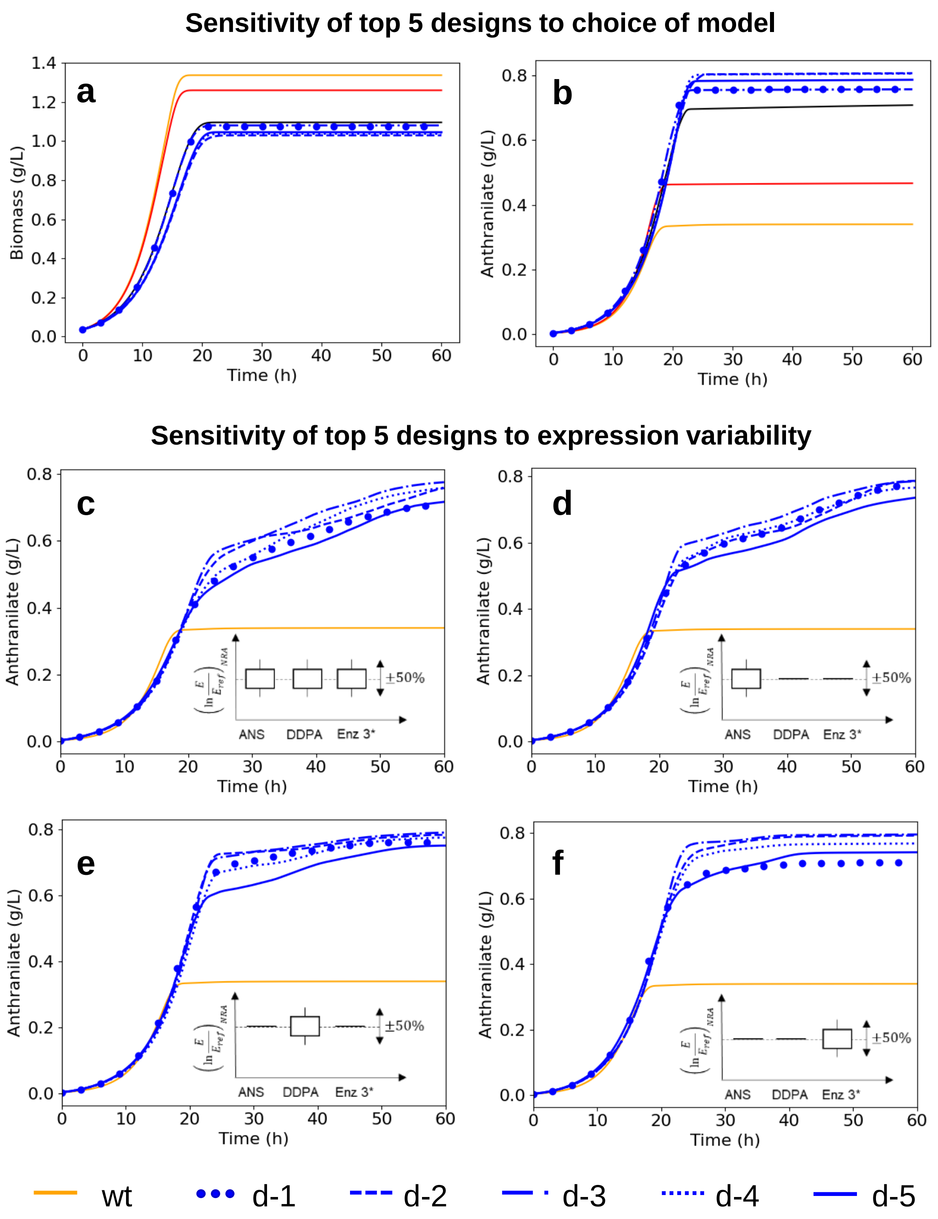

Supplementary Figure 12. Sensitivity analysis of the top 5 designs from eK_trpD9923. Mean biomass (a) and anthranilate titers (b) across models for each of the top 5 designs generated using eK_trpD9923. The designs all provide superior anthranilate titers when compared with the insilico implementations of the engineering strains, eK_trpD9923_d1 (red) and eK_trpD9923_d2 (black). The designs were sensitive to the perturbation of all 3 enzymes together (c), and ANS alone (d) but robust to incorrect implementations of DDPA (e) and the third enzyme (f). Source data are provided as a Source Data file.

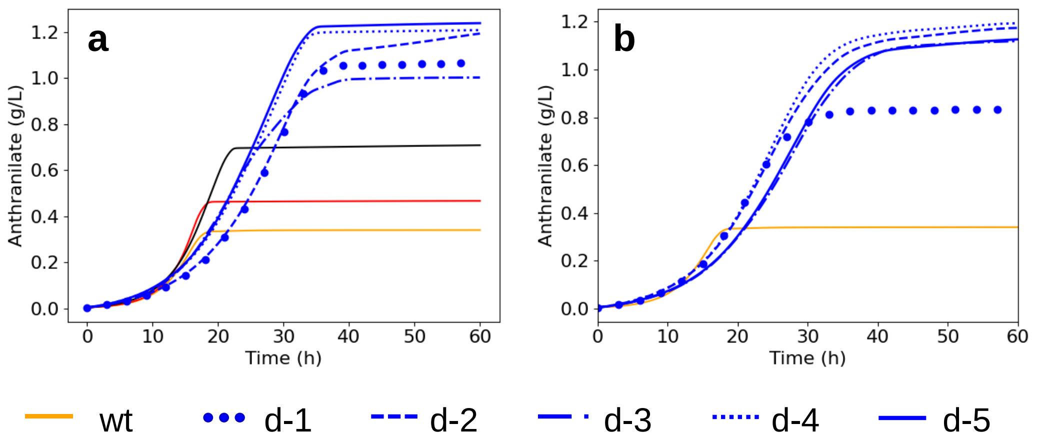

Supplementary Figure 13. Verification of the top 5 designs from eK_trpD9923_d2 in a bioreactor setting. (a) The mean anthranilate titers of the designs were superior to those of the two engineering strains, eK_trpD9923_d1 (black) and eK_trpD9923_d2 (red). (b) Four out of the five designs also proved robust to 50% perturbations applied to the NRA suggested fold changes of all enzymes simultaneously. Although d-1 (ANS, DHQS, GLUDy) provided lower titers under enzymatic perturbations, it still produced higher mean titers than the two engineering strains. Source data are provided as a Source Data file.

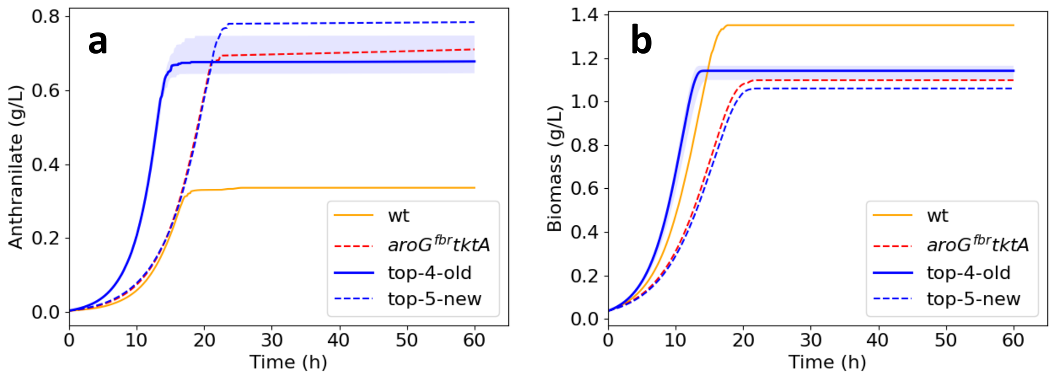

Supplementary Figure 14. Performance of top K_trpD9923 designs in eK_trpD9923. Median and interquartile range of anthranilate titers (a) and biomass (b) when the top 4 designs generated using K_trpD9923 are implemented in eK_trpD9923. Mean anthranilate and biomass curves are also provided for the reference strain eK_trpD9923 (orange), the in-silico double mutant eK_trpD9923_d2 (red dashed), and the top 5 designs from eK_trpD9923 (blue dashed). The old designs produce lower anthranilate titers than the new designs when implemented in the new models, albeit with better dynamics than the other strains. Source data are provided as a Source Data file.

Supplementary Table 1. Mean fold changes for the top 5 robust designs for improving anthranilate yield.

Design	Enzyme	Mean suggested fold-change
d-1	DDPA↑	4.15
	GLUDy↓	1.2
	PYK↑	1.57
d-2	DDPA↑	3.91
	GLUDy↓	1.19
	PGI↓	2.23
d-3	DDPA↑	3.92
	GLUDy↓	1.2
	GND↓	1.62
d-4	DDPA↑	3.89
	GLUDy↓	1.21
	HEX1↓	3.28
d-5	DDPA↑	2.48
	GLUDy↓	1.13
	ANS↑	2.87

Supplementary Table 2. Top 5 designs for eK_trpD9923 and eK_trpD9923_d2 and the mean of the suggested changes in enzyme activities across the models.

	eK_trpD9923	
Design	Enzyme	Mean suggested fold-change across 13 kinetic models
d-1	ANS↑	4.01
	DDPA↑	7.25
	FBA↓	1.39
d-2	ANS↑	4.23
	DDPA↑	7.32
	GAPD↓	1.62
d-3	ANS↑	4.16
	DDPA↑	7.25
	PGK↓	2.41
d-4	ANS↑	4.01
	DDPA↑	6.62
	G6PDH2r↑	1.22
d-5	ANS↑	3.92
	DDPA↑	6.86
	PGL↑	1.35

eK_trpD9923_d2	
Enzyme	Mean suggested fold-change across 13 kinetic models
ANS↑	8.84
DHQS↑	6.02
GLUDy↓	2.65
ANS↑	9.57
DHQS↑	6.04
AKGDH↑	2.38
ANS↑	6.99
DDPA↑	6.35
GAPD↓	4.30
ANS↑	6.28
DDPA↑	8.70
FBA↓	2.62
ANS↑	4.58
DDPA↑	7.69
PGL↑	1.84

Supplementary Table 3. Key Variables of the NRA formulation.

Type	Formula
Log flux fold change,<br>Log flux	$F_j = \ln\left(\frac{v_j}{v_{j,ref}}\right)$; $\widetilde{F_j} = F_j + \ln(v_{j,ref})$
Log concentration fold change,<br>Log concentration	$M_i = \ln\left(\frac{[x_i]}{[x_{i,ref}]}\right)$; $\widetilde{M_i} = M_i + \ln[x_{i,ref}]$
Log enzyme activity fold change – Down / Up	$E_k^D = \ln\left(\frac{[e_k]}{[e_{k,ref}]}\right)$; $E_k^U = \ln\left(\frac{[e_k]}{[e_{k,ref}]}\right)$
Enzyme up/down regulation coupling (Binary)	E_k^{UU}, E_k^{DU}
Enzymatic intervention coupling (Binary)	z_k
Standard reaction Gibb's free energy	$\Delta G_j'^0$

Supplementary Table 4. Constraints in the NRA formulation.

Type	Formulation
Flux response balance, Concentration response balance	$F_j - \sum_{k \in K}\left(C_{e_k}^{v_j} \cdot E_k^D + C_{e_k}^{v_j} \cdot E_k^U\right) = 0;\ \forall j \in \mathcal{J}$ $M_i - \sum_{k \in K}\left(C_{e_k}^{x_i} \cdot E_k^D + C_{e_k}^{x_i} \cdot E_k^U\right) = 0;\ \forall i \in \mathcal{I}$
Prohibition of simultaneous up and down enzyme regulation	$E_k^{\mathrm{UU}} + E_k^{\mathrm{DU}} \leq 1 \quad ; \forall k \in \mathcal{K}$
Binary variable coupling to magnitude of regulation	$E_k^D - \xi \cdot E_k^{DU} \leq 0$ $E_k^U - \xi \cdot E_k^{UU} \leq 0 \quad ; \ \forall k \in \mathcal{K}$
Enzyme intervention coupling	$E_K^U + E_K^D + 1000 \cdot z_k \leq 1000 \quad ; \forall k \in \mathcal{K}$
2nd law of thermodynamics	$\Delta G_{r,j}'^{0} + RT \sum_{m \in M_i} \widetilde{M}_m^j < 0 \quad ; \forall j \in \mathcal{J}$
Limit on number of enzymatic interventions to 3	$\sum_{k \in K} 1 - z_k \leq 3$
Constraints on concentration and enzymatic fold changes (3 and 5 fold)	$M_i < \ln(3)\ ;\ \forall i \in \mathcal{I}$ $E_k < \ln(5)\ ;\ \forall k \in \mathcal{K}$

Supplementary Table 5. Indices of the various variables and spaces in the NRA formulation

Index letter	Type	Refers to	Set or unit
$\boldsymbol{i}$	Index	Reaction	$i \in \mathcal{I}$
$\boldsymbol{j}$	Index	Metabolite	$j \in \mathcal{J}$
$\boldsymbol{k}$	Index	Enzyme	$k \in \mathcal{K}$
$\boldsymbol{m}$	Index	Metabolite participating in reaction i	$m \in \mathcal{M}_i$
$\boldsymbol{v_i}$	Variable	Flux of reaction i	$mmol \cdot gDW^{-1} \cdot h^{-1}$
$\boldsymbol{x_j}$	Variable	Concentration of metabolite j	M
$\boldsymbol{e_k}$	Variable	Catalytic activity of enzyme k	$mmol \cdot h^{-1}$
$\boldsymbol{\tilde{F}_i}$	Variable	Scaled flux deviation of reaction i	$\tilde{F}_i \in \mathbb{R}$
$\boldsymbol{F_i}$	Variable	Flux deviation of reaction i	$F_i \in \mathbb{R}$
$\boldsymbol{\tilde{M}_j}$	Variable	Scaled concentration deviation of metabolite j	$\tilde{M}_j \in \mathbb{R}$
$\boldsymbol{M_j}$	Variable	Concentration deviation of metabolite j	$M_j \in \mathbb{R}$
$\boldsymbol{E_k^U}$	Variable	Upregulation of catalytic activity of enzyme k	$E_k^U \in \mathbb{R}$
$\boldsymbol{E_k^D}$	Variable	Downregulation of catalytic activity of enzyme k	$E_k^D \in \mathbb{R}$
$\boldsymbol{\Delta_r G_i^{'\,o}}$	Variable	Standard Gibbs free energy change of reaction i	$\Delta_r G_i^{'\,o} \in \mathbb{R}$
$\boldsymbol{E_k^{UU}}$	Variable	Binary upregulation of catalytic activity of enzyme k	$E_k^{UU} \in \{0,1\}$
$\boldsymbol{E_k^{DU}}$	Variable	Binary downregulation of catalytic activity of enzyme k	$E_k^{DU} \in \{0,1\}$
$\boldsymbol{z_k}$	Variable	Deregulation of enzyme k	$z_k \in \{0,1\}$
$\boldsymbol{C_e^v}$	Parameter	Flux control coefficient	$C_e^v \in \mathbb{R}$
$\boldsymbol{C_e^x}$	Parameter	Concentration control coefficient	$C_e^x \in \mathbb{R}$
$\boldsymbol{v_{i,ref}}$	Parameter	Reference flux of reaction i	$mmol \cdot gDW^{-1} \cdot h^{-1}$
$\boldsymbol{x_{j,ref}}$	Parameter	Reference concentration of metabolite j	M
$\boldsymbol{e_{k,ref}}$	Parameter	Reference catalytic activity of enzyme k	$mmol \cdot h^{-1}$
$\boldsymbol{\Delta_r G_i^{'\,o}}$	Parameter	Estimated standard Gibbs free energy change of reaction i	$\Delta_r G_i^{'\,o} \in \mathbb{R}$
$\boldsymbol{R}$	Parameter	Gas constant	$R = 1.9872 \times 10^{-3} kcal \cdot K^{-1} \cdot mol^{-1}$
$\boldsymbol{T}$	Parameter	Temperature	K

Here $\mathcal{I}, \mathcal{J}, \mathcal{K}$ represent the set of indices of all the different reactions, metabolites, and enzymes in the model, and $\mathcal{M}_i$ is the set of indices corresponding to the different metabolites that participate in the reaction with index i.

Supplementary references

[1] Balderas-Hernández, V. E. *et al.* Metabolic engineering for improving anthranilate synthesis from glucose in *Escherichia coli*. *Microb. Cell Fact.* **8**, 1–12, (2009).

[2] Weilandt, D. R. *et al.* Symbolic kinetic models in python (SKiMpy): intuitive modeling of large-scale biological kinetic models. *Bioinformatics*. **39**, 2004–2006 (2023).

[3] Kwak, J. H., Hong, K. W., Lee, S. H., Hong, J. H., and Lee, S. Y. Identification of amino acid residues involved in feedback inhibition of the anthranilate synthase in *Escherichia coli. Journal of Biochemistry and Molecular Biology* **32**, 20–24 (1999).

[4] Lin, S. *et al.* Site-directed mutagenesis and over expression of aroG gene of *Escherichia coli* K-12. *Int. J. Biol. Macromol.* **51**, 915–919 (2012).

[5] Khodayari, A. and Maranas, C. D. A genome-scale Escherichia coli kinetic metabolic model k-ecoli457 satisfying flux data for multiple mutant strains. *Nat. Commun.* **7**, 13806 (2016).

[6] Dorf, R. C. and Bishop, R. H. *Modern Control Systems*, 12th ed. Prentice Hall, 2011.
