# Supplementary material for: Rational strain design with minimal phenotype perturbation
Source: Nat Commun. 2024 Jan 24;15:723. doi: 10.1038/s41467-024-44831-0 (PMC10808392; doi:10.1038/s41467-024-44831-0)
Supplement: Supplementary file 3 — Description of Additional Supplementary Files [file 41467_2024_44831_MOESM3_ESM.pdf]

File Name: Supplementary Data 1

Description : Reaction mechanisms for each of the reactions in the kinetic model

File Name: Supplementary Data 2

Description: Regulatory information for each of the regulatory interactions added in the model
