# Supplementary material for: Rational strain design with minimal phenotype perturbation
Source: Nat Commun. 2024 Jan 24;15:723. doi: 10.1038/s41467-024-44831-0 (PMC10808392; doi:10.1038/s41467-024-44831-0)
Supplement: Supplementary file 6 — Reporting Summary [file 41467_2024_44831_MOESM6_ESM.pdf]

nature portfolio

Corresponding author(s): Ljubisa Miskovic, Vassily Hatzimanikatis

Last updated by author(s): Dec 11, 2023

Reporting Summary

Nature Portfolio wishes to improve the reproducibility of the work that we publish. This form provides structure for consistency and transparency in reporting. For further information on Nature Portfolio policies, see our Editorial Policies and the Editorial Policy Checklist.

Statistics

For all statistical analyses, confirm that the following items are present in the figure legend, table legend, main text, or Methods section.

n/a	Confirmed	
☐	☒	The exact sample size (*n*) for each experimental group/condition, given as a discrete number and unit of measurement
☒	☐	A statement on whether measurements were taken from distinct samples or whether the same sample was measured repeatedly
☒	☐	The statistical test(s) used AND whether they are one- or two-sided<br>*Only common tests should be described solely by name; describe more complex techniques in the Methods section.*
☒	☐	A description of all covariates tested
☒	☐	A description of any assumptions or corrections, such as tests of normality and adjustment for multiple comparisons
☐	☒	A full description of the statistical parameters including central tendency (e.g. means) or other basic estimates (e.g. regression coefficient) AND variation (e.g. standard deviation) or associated estimates of uncertainty (e.g. confidence intervals)
☒	☐	For null hypothesis testing, the test statistic (e.g. *F*, *t*, *r*) with confidence intervals, effect sizes, degrees of freedom and *P* value noted<br>*Give P values as exact values whenever suitable.*
☒	☐	For Bayesian analysis, information on the choice of priors and Markov chain Monte Carlo settings
☒	☐	For hierarchical and complex designs, identification of the appropriate level for tests and full reporting of outcomes
☒	☐	Estimates of effect sizes (e.g. Cohen's *d*, Pearson's *r*), indicating how they were calculated

Our web collection on statistics for biologists contains articles on many of the points above.

Software and code

Policy information about availability of computer code

Data collection

A snapshot of the code, providing the exact version used in this study, is available at https://zenodo.org/doi/10.5281/zenodo.10352653

The code used to generate strain designs is publicly available at https://github.com/EPFL-LCSB/NOMAD . The scripts can be run within a docker environment to ensure reproducibility and to ensure that all the requisite packages come pre-installed. The only extra requirement is for the user to download the CPLEX solver version 12.8 from IBM and copy paste the contents into the NOMAD/docker/solvers folder. Further instructions are within the NOMAD/docker/solvers/instructions.txt file.

We used in house developed code written in python 3.6 in the form of 3 separate packages.
1. pyTFA for thermodynamics based flux analysis https://doi.org/10.1093/bioinformatics/bty499
2. SKiMPy for generating kinetic models around sampled steady state profiles of concentrations and fluxes https://pubmed.ncbi.nlm.nih.gov/36495209/
3. NRAplus for generating strain design alternatives using the chosen kinetic models. The NRA method itself has been published earlier https://pubmed.ncbi.nlm.nih.gov/33895366/ . In this work, the NRA method was implemented in Python with extensions.

The codes themselves use commercially available softwares and packages:
1. CPLEX solver suite by IBM, version 12.8 for solving the mixed integer linear problems
2. Sundials https://computing.llnl.gov/projects/sundials solver suite for integrating the ordinary differential equations. Sundials need not be installed separately. It is taken care of while building the docker environment. The corresponding version of scikits.odes that is compatible with Sundials is also installed automatically.

Data analysis

We used Python packages for data analysis and plotting.

March 2021

Data analysis

1. Pandas dataframes
2. Seaborn for plotting
3. Matplotlib for plotting

The relevant versions are all contained within the docker environment

For manuscripts utilizing custom algorithms or software that are central to the research but not yet described in published literature, software must be made available to editors and reviewers. We strongly encourage code deposition in a community repository (e.g. GitHub). See the Nature Portfolio guidelines for submitting code & software for further information.

Data

Policy information about availability of data

All manuscripts must include a data availability statement. This statement should provide the following information, where applicable:

- Accession codes, unique identifiers, or web links for publicly available datasets
- A description of any restrictions on data availability
- For clinical datasets or third party data, please ensure that the statement adheres to our policy

Source data are provided with the paper. Data extracted from the experimental work by Balderas-Hernandez et al. are available in the GitHub repository https://github.com/EPFL-LCSB/NOMAD. The data corresponding to the kinetic models consists of kinetic parameter sets in a yaml file and the corresponding steady-state data in a .csv file. The steady-states corresponding to each of the kinetic parameter sets are stated within the code.

The strain designs themselves can be generated by running the codes, while the major designs are all reported in the paper. The average proposed values of enzyme regulation of the top 5 designs is reported in the supplementary material. The parameters characterizing the final set of 10 kinetic models, along with the final list of 41 unique designs are provided under NOMAD/anthranilate-study/data.

Human research participants

Policy information about studies involving human research participants and Sex and Gender in Research.

Reporting on sex and gender	n/a
Population characteristics	n/a
Recruitment	n/a
Ethics oversight	n/a

Note that full information on the approval of the study protocol must also be provided in the manuscript.

Field-specific reporting

Please select the one below that is the best fit for your research. If you are not sure, read the appropriate sections before making your selection.

☒ Life sciences ☐ Behavioural & social sciences ☐ Ecological, evolutionary & environmental sciences

For a reference copy of the document with all sections, see nature.com/documents/nr-reporting-summary-flat.pdf

Life sciences study design

All studies must disclose on these points even when the disclosure is negative.

Sample size	All performed optimizations are deterministic, sample size is irrelevant. Regarding Figures 5b and 6a,b the data are taken from the experimental work by Balderas-Hernandez et al.
Data exclusions	No data were excluded.
Replication	All performed optimizations are deterministic, results are reproducible. Replication is not applicable.
Randomization	All performed optimizations are deterministic. Randomization is not applicable.
Blinding	All performed optimizations are deterministic. Blinding is not applicable.

Reporting for specific materials, systems and methods

We require information from authors about some types of materials, experimental systems and methods used in many studies. Here, indicate whether each material, system or method listed is relevant to your study. If you are not sure if a list item applies to your research, read the appropriate section before selecting a response.

Materials & experimental systems

n/a	Involved in the study
☒	☐ Antibodies
☒	☐ Eukaryotic cell lines
☒	☐ Palaeontology and archaeology
☒	☐ Animals and other organisms
☒	☐ Clinical data
☒	☐ Dual use research of concern

Methods

n/a	Involved in the study
☒	☐ ChIP-seq
☒	☐ Flow cytometry
☒	☐ MRI-based neuroimaging
